# The effectiveness and adaptiveness of suppressing unwanted thoughts

**DOI:** 10.3758/s13423-025-02763-w

**Published:** 2025-12-09

**Authors:** Aneta Niczyporuk

**Affiliations:** Faculty of Philosophy and Cognitive Science, University of Bialystok, ul. Świerkowa 20 B, 15-328 Białystok, Poland

**Keywords:** Thought suppression, Memory suppression, Memory control, *White bear* paradigm, *Think/no-think* paradigm, Intrusions, Intrusive thoughts

## Abstract

The status of thought suppression in contemporary psychology remains ambiguous. The literature contains claims both about its counterproductive consequences and about its potential utility. The aim of this article is to review and evaluate evidence concerning the effectiveness and adaptiveness of thought suppression across different research traditions. The first part of the paper examines effectiveness. Studies from the two main paradigms—the *white bear* and the *think/no-think* paradigms—are compared. Findings suggest that, in healthy populations, suppression can be effective, while paradoxical effects have not been convincingly demonstrated. The second part addresses adaptiveness. Questionnaire studies, clinical observations, and (quasi-)experimental research are discussed. This body of evidence indicates that thought suppression may be adaptive, depending on factors such as individual differences and context. Finally, potential desirable and undesirable suppression outcomes are discussed.

## Introduction

A lack of consensus in psychology is more the rule than the exception. Disputes among psychologists, however, typically concern the conceptualization and underlying mechanisms of mental phenomena, rather than the basic findings about those phenomena. Against this backdrop, the issue of thought suppression stands out. Here, the debate extends not only to the processes underlying suppression, but also to whether it is effective or paradoxical in its outcomes and, consequently, whether it is adaptive or maladaptive.

On the one hand, more than a century ago Janet and Freud recommended confronting uncomfortable content rather than avoiding it (Rubenstein et al., 2024). Similarly, today the conviction that suppressing thoughts is both ineffective and detrimental is widely held, especially among clinical psychologists (e.g., Aldao et al., 2010; Ehlers & Clark, 2000; Hiller et al., 2019; M. R. Smith et al., 2023). This view is supported by the *white bear* (WB) studies, in which paradoxical effects of thought suppression have been repeatedly observed (for recent meta-analysis, see D. A. Wang et al., 2020), as well as by a large body of questionnaire-based research indicating associations between thought suppression and the occurrence of mental disorders (e.g., Genuchi et al., 2025; Jaso et al., 2020; K. E. Smith et al., 2019; Wegner & Zanakos, 1994). Consequently, the assumption of the harmfulness of thought suppression has been incorporated into psychological practice (e.g., Todd & Branch, 2022; Wenzel, 2021).

At the same time, however, a parallel line of investigation—centered around the *think/no-think* (TNT) paradigm—has been intensively developed (Anderson & Green, 2001; Nardo & Anderson, 2024). TNT research has quite consistently demonstrated effective thought control in healthy population (for meta-analysis, see C. Clark et al., 2025) and has provided substantial neuroimaging evidence regarding the brain mechanisms underlying thought suppression (for review, see Anderson et al., 2025). Based on these findings, particularly among cognitive psychologists, suppression has been regarded as potentially effective and adaptive (e.g., Engen & Anderson, 2018; Fawcett & Hulbert, 2020; Nørby, 2018).

The aim of this paper is to compile and evaluate the arguments for and against the effectiveness and adaptiveness of thought suppression emerging from different research traditions. Here, I understand thought suppression as deliberate attempts either to get rid of unwanted content from consciousness (Purdon, 2020; Rassin, 2005) or to prevent it from entering consciousness. Importantly, for attempts to block unwanted content to be classified as thought suppression, they must occur during moments of heightened risk of an unwanted thought intruding into consciousness, or immediately following such an intrusion.

This is a broad definition, as in principle the goal of eliminating content from consciousness can be pursued via different mechanisms (e.g., direct suppression and thought substitution; see the next section). While the effects of these mechanisms may differ, the primary purpose of this paper is to determine whether it can be worth attempting to suppress thoughts at all. Questions of how (and when) suppression should be applied arise in the next step.

At the same time, the above definition narrows the phenomenon of thought suppression to the immediate management of unwanted thoughts. This approach, I hope, captures what is prototypical for this term without extending it to other phenomena that may, but do not necessarily, relate to it. Therefore, I will not consider as thought suppression prospective actions aimed at lowering the likelihood of unwanted thoughts in the future, such as avoiding situations and stimuli that may evoke them (as in broader construct of cognitive and behavioral avoidance Ehlers & Steil, 1995; Martell & Puspitasari, 2023; cf. Catarino et al., 2015), deciding to forget a recently encountered situation or material (as in directed forgetting paradigm; Macleod, 1999), punishing oneself for experiencing intrusions (Wells & Davies, 1994), or even undergoing psychotherapy. Regarding the term intrusions, it is used here to broadly denote conscious, involuntary, unwanted thoughts, while acknowledging this is not a precise definition (Visser et al., 2021).

The article is divided into two main parts. In the first part, I present research on the effectiveness of thought suppression. These studies have been conducted within two paradigms: the WB paradigm and the TNT paradigm. In light of the available data, I conclude that there is potential for effective thought suppression in healthy populations, and that the evidence for paradoxical effects of suppression is insufficient—despite their still being often regarded as an established scientific fact (Purdon, 2020).

In the second part, I address the adaptiveness of thought suppression. I refer to questionnaire studies, clinical practice, as well as experimental and quasi-experimental research. Based on these analyses, I argue for the need to relativize claims about the adaptiveness of thought suppression with respect to the individual and the context. Thought suppression should not be assumed to be generally adaptive or maladaptive; instead, we should specify the conditions of its adaptiveness. I briefly discuss several such proposed conditions.

The paper ends with a synthesis of the reviewed evidence and the conclusions derived from its analysis.

## (In?)effectiveness of thought suppression

Before addressing the question of the adaptiveness of thought suppression, it is essential to establish whether it can, in fact, be effective. Although some evidence in this regard comes from clinical observations (to be discussed later in the text), the present section focuses on basic experimental research, which enables the examination of causal relationships and their underlying mechanisms. Research on the effectiveness of thought suppression centers around two paradigms: the WB paradigm and the TNT paradigm. In the following sections, both paradigms are presented and then compared. Table 1 summarizes the key points of the analysis.

**Table 1:** Summary of Key Findings on Thought Suppression Effectiveness

Key Findings on Thought Suppression Effectiveness	
Short-term effectiveness (during suppression)	
White Bear research Thought suppression is effective; under cognitive load a paradoxical increase in the avoided content occurs (immediate enhancement effect).	Think/No-Think research Thought suppression is effective in healthy populations.
Long-term effectiveness (after suppression)	
White Bear research Thought suppression is counterproductive: a paradoxical increase in avoided content is observed (rebound effect). Replication is insufficient, and publication bias is likely.	Think/No-Think research Thought suppression is effective in healthy populations: poorer memory for previously suppressed content is observed (suppression-induced forgetting), with good replicability.

### *White bear* paradigm

In one of his works, Fyodor Dostoevsky expressed the belief in the ineffectiveness of thought suppression by giving an example of trying to avoid thinking about a polar bear, which would paradoxically result in the animal's constant presence in the mind (Dostoyevsky, 1985, p. 62). About 100 years later, Dostoevsky’s example inspired the development of the first experimental paradigm on thought suppression, known as the WB paradigm (Wegner et al., 1987).

**Fig. 1:**
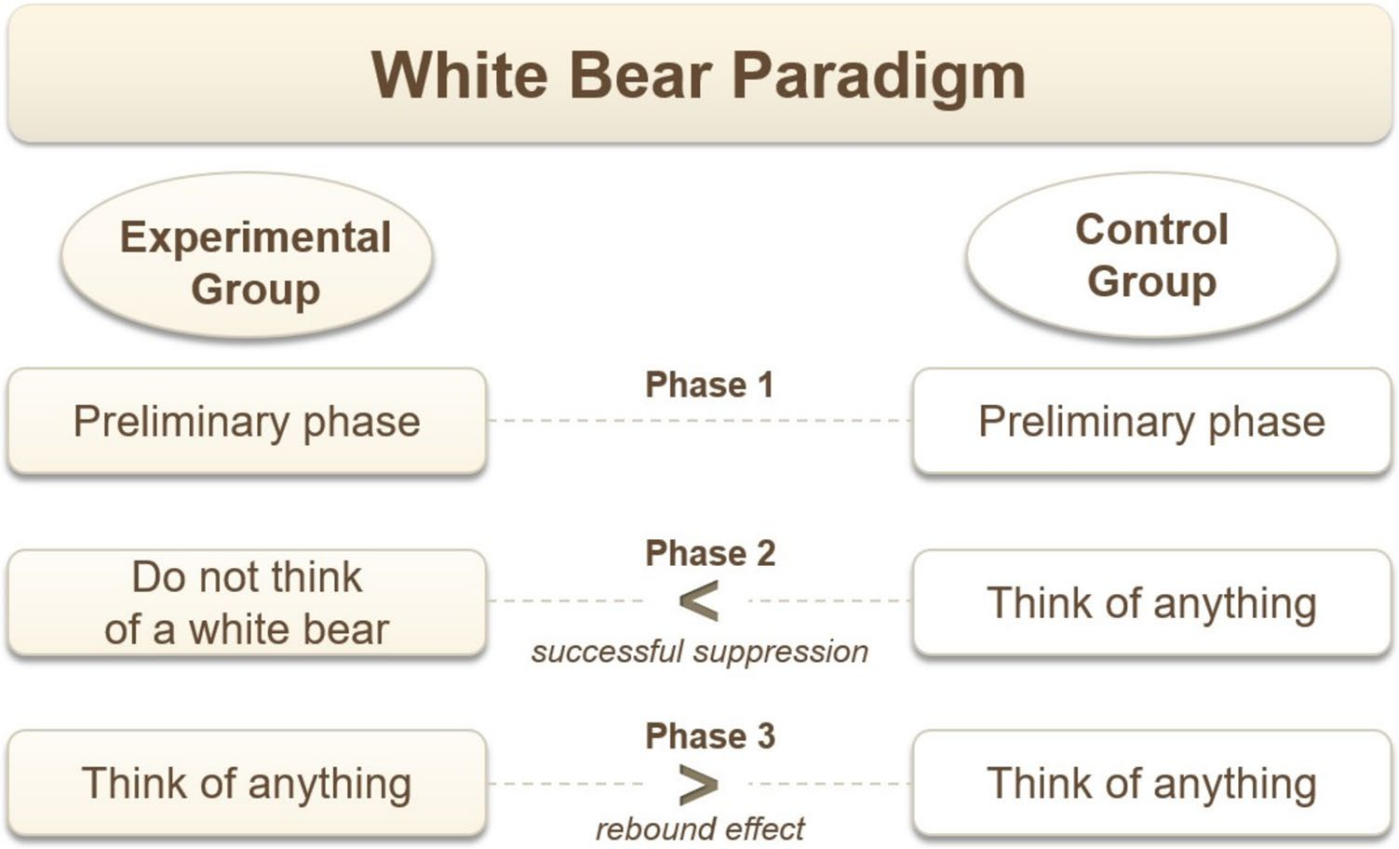
Typical course and results of an experiment using the White Bear paradigm. In the second phase, the experimental group shows fewer target thoughts than the control group (as illustrated), unless a cognitive load is introduced, which leads the experimental group to think more about the target. In the third phase, the rebound effect is usually observed - that is, the experimental group reports more target thoughts than the control group, regardless of cognitive load

In a standard study of the WB paradigm, we have two groups of participants who are simply asked to think during the several minutes they spend in the laboratory. The participants are also instructed to signal whenever the target content, such as a thought about the WB, occurs. During one phase, the experimental group is additionally asked to suppress the very thought. In the following phase, all participants are allowed to think about anything they like (see Fig. 1). The frequency of target thoughts in each phase in each group is analyzed.

Regarding the short-term effectiveness of suppression (i.e., its effectiveness during efforts to avoid mental content), the WB studies indicate that thought suppression is effective. In the experimental group, there are usually fewer target thoughts in the suppression phase than in the comparison phase in the control group (for meta-analysis, see Abramowitz et al., 2001; Magee et al., 2012; D. A. Wang et al., 2020). Although there are reports of the immediate enhancement effect, i.e., an increase in the frequency of target thoughts during thought suppression (e.g., Salkovskis & Campbell, 1994), research tends to observe a reversal of the effect (e.g., Abramowitz et al., 2001). It seems that the immediate enhancement effect may occur when thought suppression is attempted under cognitive load (D. A. Wang et al., 2020). In other cases suppression should be effective in the short run.

As far as the long-term consequences of thought suppression are concerned, the most commonly obtained result in the WB research is the rebound effect, which is the more frequent occurrence of the target thought in the experimental group after the suppression phase compared with the control group, which did not attempt suppression (for review, see Wenzlaff & Wegner, 2000). The rebound effect was found in studies using a variety of target content, ranging from WB thoughts (e.g., Wegner et al., 1987), through emotionally laden content (e.g., Davies & Clark, 1998), to specific content such as stereotypical thoughts (e.g., Macrae et al., 1994, 1996), painful sensations (e.g., Cioffi & Holloway, 1993), or thoughts about food (e.g., Erskine & Georgiou, 2010). Similar paradoxical effects of self-control also appeared in WB paradigm-inspired research on the control of movements or emotions (for review, see Wegner, 2009). In sum, the long-term paradoxical effects of thought suppression was revealed in a number of studies utilizing a variety of stimuli.

The paradoxical effects of thought suppression are typically explained by the theory of ironic processes of mental control (Wegner, 1994; Wegner & Erber, 1992; Wenzlaff & Wegner, 2000). This theory posits two processes involved in thought control: an effortful operating process that actively searches for topics to occupy consciousness, and an effortless monitoring process that scans for signs of unwanted content intruding into consciousness. As long as the operating process is engaged, attention can be successfully diverted away from the unwanted thought. However, when it ceases—either due to the cessation of mental effort to avoid the content or a lack of cognitive resources to sustain the process—the rebound effect is expected. This ironic rebound arises because the monitoring process, by maintaining vigilance for the unwanted content, paradoxically keeps it highly accessible. Thus, Wegner’s theory accounts for both the rebound effect and the immediate enhancement effect under cognitive load.

Nevertheless, it is not the only explanation for findings in the WB paradigm. Paradoxical effects may also result from the formation of new, negation-based associations between the unwanted content and previously distracting contents, which later become retrieval cues (Wegner et al., 1987; Wenzlaff & Wegner, 2000). Additionally, increases in the frequency of prohibited thoughts have been interpreted as a consequence of participants’ psychological reactance (Wallaert et al., 2023; Wenzlaff & Wegner, 2000). Finally, according to the motivational inference model (Förster & Liberman, 2004; Liberman & Förster, 2000), paradoxical effects arise from participants’ inferred motivation to think about the target content. During the thought suppression phase, participants infer that they apparently must want to think about the unwanted content, since it sometimes appears in their consciousness despite their efforts to avoid it. This inferred motivation leads to increased thoughts about the target content in the subsequent phase, when suppression is relaxed, thereby producing the rebound effect. Indeed, the motivational inference model explains certain findings that are difficult to reconcile with the dominant ironic processes account (e.g., Denzler et al., 2010; Koole & van Knippenberg, 2007; Liberman & Förster, 2000).

Actually, it is difficult to identify a single theory that comprehensively explains all available data from the WB task, largely due to the high inconsistency of results. Although three meta-analyses of WB studies confirmed the existence of the rebound effect, with its magnitude estimated as small to medium (Abramowitz et al., 2001; Magee et al., 2012; D. A. Wang et al., 2020), substantial number of individual experiments have failed to observe it (for review, see Purdon, 2020). For example, data from these meta-analyses indicate that the rebound effect was numerically reversed in 18% (Abramowitz et al., 2001) to 46% (D. A. Wang et al., 2020) of the analyzed cases.^1^ Such statistics may suggest that the rebound effect is an artifact arising from publication bias. This interpretation is further supported by Wang and colleagues’ meta-analysis (D. A. Wang et al., 2020), which detected a small study bias. Notably, after correcting for this bias, the rebound effect ceased to be statistically significant. Interestingly, the (inverted) enhancement effect remained significant even after this correction.

The risk of publication bias influencing reports of the rebound effect is substantial. However, this is not the only potential explanation for the effect’s poor replicability. Methodological concerns also warrant consideration. The primary indicator of thought frequency in the WB procedure relies on introspection, which renders it vulnerable to potential confounders. The number of reported target thoughts may be affected by participants’ motivation to perform well or by reactance. Furthermore, participants may vary in how they interpret instructions to signal the occurrence of specific mental content. For example, it remains unclear whether “thinking about a white bear” includes detailed thoughts about the animal’s features, such as its teeth, or fleeting thoughts of the instruction mentioning the WB (for a review of methodological concerns, see Purdon, 2020).

To mitigate these issues, some WB studies incorporated more objective measures, such as the lexical decision task, to estimate the activation levels of target content (e.g., Giuliano & Wicha, 2010; Logel et al., 2009; Tolin et al., 2002). While such studies typically confirm the paradoxical effects, they often do not assess whether these alternative measures correlate with self-reported frequency of target thoughts (e.g., Logel et al., 2009). When correlations are examined, the expected relationships are not consistently observed (e.g., Berry et al., 2007; D. A. Wang et al., 2017). Consequently, it remains uncertain what these measures truly capture.

In summary, conclusions drawn from studies using the WB paradigm should be interpreted with caution. The rebound effect demonstrates poor replicability and is likely influenced by publication bias. Additionally, the reliability of the procedure’s methodology has not been sufficiently scrutinized, which may contribute to the mixed findings. On a more positive note, effects observed during the suppression phase appear to be somewhat more consistent (D. A. Wang et al., 2020).

### *Think/No-Think* paradigm

**Fig. 2:**
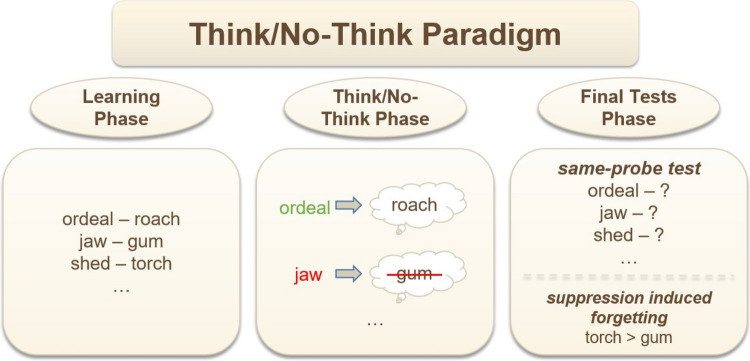
Typical course and results of an experiment using the Think/No-Think paradigm. In the learning phase, participants memorize word pairs. In the Think/No-Think phase, cue words from most pairs are presented, and participants are instructed either to think of the target word (i.e., the second word in the pair, e.g., “roach”) or to avoid thinking about it (“gum”). Baseline words are not prompted during this phase (“torch”). In the final tests phase, participants attempt to recall all previously learned target words. In the same-probe test, they are again presented with the original cue words (as illustrated), whereas in the independent-probe test they are given a new cue and the first letter of the target word (e.g., “insect – r…?”). The results typically show suppression induced forgetting, that is, poorer memory for previously suppressed

Alongside the WB paradigm, another approach to studying thought suppression is the TNT paradigm (Anderson & Green, 2001). These procedures differ substantially and lead to contradictory conclusions. The TNT procedure begins with participants learning several dozen item pairs, most commonly words. In the subsequent phase, most of the first items from the learned pairs (*cue items*) are presented. Participants are instructed, in response to each cue item, to either recall the second item from the pair (*think item*) or to actively suppress thoughts about the second item (*no-think item*). At the end of the experiment, an unexpected memory test requires recalling the second item from each pair (*target items*). In this memory test, no-think items are not only remembered worse than think items but also worse than baseline items—items that were presented during the learning phase but were not prompted during the TNT phase (see Fig. 2). This effect, known as suppression-induced forgetting, suggests our ability to effectively and actively suppress thoughts.

Suppression-induced forgetting demonstrates the long-term effectiveness of thought suppression, as participants recall previously suppressed content less accurately. Research tracking progress in avoiding no-think items during the TNT phase has also confirmed the short-term effectiveness of suppression: with repeated attempts to suppress specific content, that content appears less frequently in consciousness (e.g., Gagnepain et al., 2017; Harrington et al., 2021).

The effectiveness of thought suppression in the TNT procedure has also been confirmed in neuroimaging studies (for reviews, see Anderson et al., 2025; Anderson & Hulbert, 2021). According to this research, suppression is initiated by a signal from the prefrontal cortex (specifically, the right dorsolateral and ventrolateral prefrontal cortices). In response to this signal, hippocampal activity, which is associated with memory retrieval, is down-regulated below baseline levels (e.g., Depue et al., 2007). The prefrontal cortex is interpreted as sending inhibitory commands to prevent the recollection of unwanted content, which is achieved through systemic hippocampal suppression. Indeed, the activity of these two structures during no-think trials predicts participants’ performance on final memory tests (e.g., Apšvalka et al., 2022; Benoit & Anderson, 2012).

This neuroimaging evidence provides strong support for the suppression-induced forgetting mechanism proposed by Anderson and colleagues (e.g., Anderson & Green, 2001; Anderson & Hulbert, 2021). According to this account, during no-think trials, the unwanted content is actively inhibited—via mechanisms partly overlapping with those engaged in the inhibition of prepotent motor responses.

Importantly, it is believed that it is the content as such, not merely its association with the cue, that is inhibited. This is demonstrated by performance on the memory tests used in the final phase of the TNT task. Typically, two types of tests are employed: *same-probe* and *independent-probe* tests. In the same-probe test, participants are presented with the original cue items and asked to recall the corresponding target items. In the independent-probe test, new cues—previously unseen but associated with the target items—are presented instead. The suppression-induced forgetting is usually observed in both test types (e.g., C. Clark et al., 2025), although the effect in the independent-probe test is less robust (cf. Wessel, 2024). Whereas forgetting in the same-probe test may reflect a weakened cue–target association, reduced recall in the independent-probe test is more consistent with the idea that the unwanted content itself is inhibited.

Results from implicit memory tests also support this hypothesis (e.g., Gagnepain et al., 2014; Hertel et al., 2018). Moreover, reports on the so-called amnesic shadow suggest that during no-think trials there may be a general suppression of processes involved in the retrieval or consolidation of mental content (for reviews, see Anderson & Subbulakshmi, 2024; Hulbert et al., 2017). Specifically, when additional, task-unrelated stimuli are presented during the TNT phase, their occurrence in close temporal proximity to no-think trials results in poorer later recall of these stimuli (e.g., Hulbert et al., 2016).

It should be noted that the mechanism of suppression-induced forgetting described above applies primarily to studies in which participants receive direct suppression instructions—namely, they are asked to suppress thoughts about the no-think item without replacing it with any other thought. Research has also examined the strategy of thought substitution, in which a thought about the no-think item is blocked by maintaining another, substitute thought in consciousness. In this case, forgetting can be explained by interference between the no-think and substitute items, potentially accompanied by an inhibitory process (e.g., Benoit & Anderson, 2012; Clark et al., 2025).

Whether using direct suppression or thought substitution, suppression-induced forgetting has been replicated numerous times (for reviews, see Anderson & Huddleston, 2012; Anderson & Hulbert, 2021; Levy & Anderson, 2008), with the effect being stronger when more no-think trials are performed (C. Clark et al., 2025; Gagnepain et al., 2017; Hanslmayr et al., 2009) and persisting even when participants were financially rewarded for each correctly recalled item (Anderson & Green, 2001; Hulbert & Anderson, 2018). To date, two meta-analyses of TNT studies have been conducted (C. Clark et al., 2025; Stramaccia et al., 2021), both confirming a small-to-moderate suppression-induced forgetting effect in healthy participants. Importantly, however, the effect was not significant in the combined cluster of clinical and subclinical samples (Stramaccia et al., 2021).

Some studies have also failed to detect suppression-induced forgetting in healthy populations (e.g., Bulevich et al., 2006; Hertel & Mahan, 2008; Meier et al., 2011; Wessel et al., 2020; Wessel, Lehmann, et al., 2025a, 2025b; Wiechert et al., 2023). Nevertheless their proportion is notably smaller than the proportion of null results in WB research (Nardo & Anderson, 2024). Based on the meta-analytic statistics, the risk of publication bias in TNT studies also appears less severe (C. Clark et al., 2025; Stramaccia et al., 2021). In the meta-analysis by C. Clark and colleagues (2025) most applied tests did not indicate publication bias, whereas in the meta-analysis by Stramaccia and colleagues (2021) some rightward asymmetry was observed in the healthy sample’s funnel plot, although this was not confirmed by Egger’s test.

Of course, even for genuine effects, a certain proportion of results will fail to reach significance. In the case of TNT, additional factors may further reduce the likelihood of detecting suppression-induced forgetting. These include the length of the experiment, participant fatigue and willingness to cooperate, individual differences, as well as experimenter behavior and stimulus selection, all of which appear to influence the magnitude and presence of the effect (Nardo & Anderson, 2024; Stramaccia et al., 2021).

Further insight into TNT effects replicability is likely to emerge in the near future from an ongoing multi-site registered replication project (Fawcett et al., 2023). Nonetheless, the available evidence suggests that suppression-induced forgetting is relatively well replicable, supporting the effectiveness of thought suppression.

### White Bear vs. Think/No-Think research

As can be seen, the two main thought suppression paradigms yield contrasting results: WB research points to paradoxical consequences of suppression, whereas TNT research indicates effective thought control. Several substantial procedural differences between the two tasks can be identified. Obvious examples include the number of contents participants are instructed to avoid (a single item in the WB vs. about a dozen in the TNT task), the duration of uninterrupted suppression (several minutes vs. a few seconds), and the indices used to measure intrusions (real-time signaling vs. memory tests). Post-suppression performance in the WB task also appears more influenced by participants’ willingness to think about the previously prohibited content (Wallaert et al., 2023), whereas this factor seems less relevant in the TNT task (e.g., Anderson & Green, 2001).

Furthermore, in the TNT phase, suppressed contents are not mentioned directly, whereas in the WB task, the target content is explicitly stated in the instructions for each phase. According to Anderson and Huddleston (2012), this is WB paradigm’s most problematic feature. Their goal-integration theory proposes that including the prohibited content in the task instructions makes suppression inherently difficult: Participants must both avoid thinking about a given item and remember not to think about it. In the TNT task, this problem does not arise because participants are simply told not to think about the item associated with a cue, without being prompted with the target item itself.

While directly mentioning the target content does strongly activate it, this should not necessarily disqualify the WB task, as such situations resemble everyday encounters with intrusive thoughts, where we often know exactly what we want to avoid thinking about. This does not inevitably lead to unsuccessful suppression. First, the repeatedly replicated reversed enhancement effect suggests that WB task participants can indeed avoid thinking about content explicitly named in the instructions. Second, TNT studies show that the occurrence of no-think thought can trigger processes leading to its forgetting (e.g., Benoit et al., 2015; Levy & Anderson, 2012). In such cases, participants know precisely which thought to avoid, yet succeed in doing so. Nonetheless, avoiding explicit reference to the unwanted content might facilitate suppression.

Despite many differences, the WB and TNT tasks also share common features. One is the partial overlap in brain regions activated during suppression phases in both paradigms (Anderson et al., 2025; Mitchell et al., 2007). Intrusion frequency in WB’s suppression phase also correlates with that observed during TNT’s no-think trials (at least during TNT practice phase; Niczyporuk & Nęcka, 2024). Findings from both paradigms further converge in showing that suppression is generally effective during the suppression phase: in the WB paradigm, this is reflected in the reversed enhancement effect (D. A. Wang et al., 2020), and in the TNT paradigm, by a decline in intrusion frequency across successive no-think trials (e.g., Levy & Anderson, 2012).

The main challenge lies in post-suppression phases. Discrepancies here may stem from the procedural differences outlined above. However, the first issue to consider is the reliability of each paradigm. As noted, suppression-induced forgetting replicates well, whereas the rebound effect is often absent. Consequently, before exploring in detail why TNT and WB research produces divergent results, it is necessary to determine the robustness of the rebound effect.

In conclusion, studies using the TNT paradigm replicate more consistently than those using the WB paradigm. They also employ more reliable and objective measures, including neuroimaging techniques. As a result, the conclusions drawn from TNT research should be considered stronger, and thought suppression itself as potentially effective in healthy population.

## (In?)adaptiveness of thought suppression

The beliefs on the effectiveness of thought suppression impact the beliefs on its adaptiveness. If thought suppression were paradoxical or fruitless, it would logically be seen as a counterproductive method of mental control or unnecessary effort. The conviction of maladaptiveness of thought suppression is widespread among clinical psychologists, who have constant contact with patients trying to get rid of intrusive memories or obsessions but are haunted by them anyway. A large body of correlational studies support this view, showing relationship between psychopathologies and thought suppression questionnaires. However, experimental and quasi-experimental studies on adaptiveness of thought suppression are lacking but few available research suggests psychological benefits coming from the ability to suppress thoughts. In the following sections, I review these three sources of data on the adaptiveness of thought suppression, starting from questionnaire research. Table 2 presents a summary of the findings. The conclusion drawn from this review is that thought suppression may be adaptive, depending on specific factors. A proposed set of such factors, along with potential beneficial and detrimental outcomes of suppression, is discussed in the final section.

**Table 2:** Summary of Key Findings on Thought Suppression Adaptiveness

Key Findings on Thought Suppression Adaptiveness
Questionnaire studies
• White Bear Suppression Inventory (WBSI) – designed to assess thought suppression propensity – is associated with psychopathology. • WBSI has a heterogeneous structure, with two frequently observed factors: intrusions and suppression. Studies separating these factors generally show no link between the suppression factor and psychopathology. • Measures of effective thought suppression correlate negatively with WBSI scores and psychopathology symptoms.
Clinical observations
• Effective treatment of patients experiencing persistent intrusions involves interventions based on exposure to distressing content. • For individuals who have just experienced a traumatic event, exposure to those memories may be harmful; interventions that engage the patient’s cognitive resources are recommended to disrupt memory consolidation. • Thought suppression mechanisms are likely engaged in psychological interventions that do not explicitly refer to suppression.
Experimental and quasi-experimental studies
• TNT studies show successful suppression of emotional thoughts in healthy populations. • The first study on thought suppression training indicates beneficial health outcomes (Mamat & Anderson, 2023). • Studies indicate difficulties in effectively suppressing thoughts among patients experiencing intrusions.

### Questionnaire-based studies on thought suppression and psychopathology

The first and most frequently used questionnaire developed to measure the tendency to suppress thoughts is the White Bear Suppression Inventory (WBSI; Wegner & Zanakos, 1994). As the name suggests, it was inspired by WB research and reports of the rebound effect. The WBSI has been widely employed to examine the relationship between thought suppression tendencies and the occurrence of psychopathology. Consequently, numerous studies have reported associations between WBSI scores and symptoms of depression (e.g., Amstadter & Vernon, 2008; Genuchi et al., 2025; Murray et al., 2021; Pegram et al., 2017; Wegner & Zanakos, 1994), anxiety (e.g., Amstadter & Vernon, 2008; Erskine et al., 2007; Wegner & Zanakos, 1994; Wilson et al., 2025), posttraumatic stress disorder (PTSD; e.g., Amstadter & Vernon, 2008; Garland & Roberts-Lewis, 2013; Lee et al., 2015; Vázquez et al., 2008), obsessive-compulsive disorder (OCD; e.g., McLaren & Crowe, 2003; Shemrani et al., 2025; Wegner & Zanakos, 1994), eating disorders (Collins et al., 2014; Ferreira et al., 2015; Lavender et al., 2015; K. E. Smith et al., 2019), borderline personality disorder (Rosenthal et al., 2005), rumination (Erskine et al., 2007; Yapan et al., 2022), drug craving (Garland & Roberts-Lewis, 2013), gambling (Riley, 2014), pain severity (Pegram et al., 2017), and lower well-being (MacDonald & Baxter, 2017; Wasylkowska et al., 2025). Questionnaires developed based on the WBSI have also shown correlations with other psychological problems (Efrati, 2019; Efrati et al., 2021; Seligowski et al., 2016). The abundance and consistency of these studies might suggest that thought suppression is highly harmful. However, this conclusion should be approached with caution for two reasons.

Firstly, the studies in question employed a correlational model. This would not pose a substantial concern if experimental studies clearly demonstrated that thought suppression leads to an increase in intrusions. However, this is not the case, and therefore, we cannot conclude that thought suppression causes or exacerbates mental health problems. It is equally plausible that individuals who suffer from recurring intrusions may be more inclined to avoid them, thus more frequently employing thought suppression.

The second problem with the presented studies is the WBSI questionnaire itself. Although designed to measure the propensity for thought suppression, only part of its items directly address attempts to avoid unwanted content; the rest pertain to the experience of intrusions. Rassin (2003) suggested that the WBSI measures unsuccessful attempts at thought suppression rather than the use of this self-regulation method per se. Further analysis by the author revealed a bifactorial structure within the questionnaire, comprising an intrusion scale and a thought suppression scale. Many other studies have also identified a two- or three-factor structure (including intrusion, suppression, and distraction scales) in the WBSI (Blumberg, 2000; Cichoń et al., 2020; Duarte et al., 2022; Höping & de Jong-Meyer, 2003; Kennedy et al., 2016; Luciano et al., 2006; Nosen & Woody, 2013; Schmidt et al., 2009; but see also research that confirmed 1-factor structure: Muris et al., 1996; Spinhoven & Van Der Does, 1999; Wegner & Zanakos, 1994).

Due to the heterogeneity of the WBSI, studies have emerged that examine the relationships between psychological problems and the intrusion scale and the thought suppression scale separately. In the work of Schmidt and colleagues (2009), only the intrusion scale predicted depression and increased anxiety, while the suppression scale showed no relationship. Similarly, Kennedy and colleagues (2016) found that, in an adolescent sample, the intrusion scale predicted anxiety-related disorders as well as obsessive-compulsive and depressive symptoms, whereas the thought suppression scale negatively predicted generalized anxiety disorder. Rassin (2003) reported mixed findings: the intrusion scale predicted anxiety and general psychopathology in a non-clinical sample, as well as obsessive-compulsive symptoms in both non-clinical and clinical samples, whereas the suppression scale predicted depressive symptoms in the non-clinical sample and general psychopathology in the clinical sample.

Nosen and Woody (2013) adapted the WBSI to focus on unwanted thoughts related to smoking cravings. They found that the intrusion scale correlated with smoking cravings and negative affect, whereas the suppression scale did not. Additionally, individuals who attempted to quit smoking but failed had higher intrusion scale scores compared to those who succeeded, with no significant difference in suppression scale scores between these groups.

Duarte and colleagues (2022) investigated the relationships between the WBSI and dissociation. Again, only the intrusion scale, not the suppression scale, was related to the scale of dissociation experiences. Finally, Watkins and Moulds (2009) studied the relationship between rumination and thought suppression. In their model, rumination was the main predictor of the WBSI suppression scale. However, the predictor of rumination was the intrusion scale, not the thought suppression scale.

As can be seen, studies examining the relationships between psychological problems and the WBSI intrusion scale and thought suppression scale separately tend to indicate no associations with the latter. This suggests that correlations between symptoms of mental disorders and the overall WBSI scores presented earlier are driven by the intrusion scale. It is important to note that the intrusion and thought suppression scales of the WBSI are correlated (e.g., Schmidt et al., 2009), which may stem from individuals experiencing more unwanted thoughts having more opportunities for thought suppression. Therefore, when investigating the relationship between thought suppression and psychopathologies, it is crucial to control for the number of experienced intrusions. Otherwise, any observed relationship may be attributed to the frequency of unwanted thoughts rather than the tendency to suppress them (cf. Höping & de Jong-Meyer, 2003; Luciano et al., 2006; Rodríguez et al., 2008).

To address limitations of the WBSI, the Thought Suppression Inventory-Revised (TSI-R) was developed (Rassin, 2003; van Schie et al., 2016). This tool consists of three scales: the intrusion scale, suppression attempts scale, and effective suppression scale. Importantly, the effective suppression scale negatively correlates with WBSI scores, confirming the hypothesis that the WBSI measures failures in thought suppression. Furthermore, the TSI-R’s intrusion and suppression attempts scales are positively correlated with each other as well as with PTSD and OCD symptoms (although the shared variance between the scales was not controlled), whereas the effective suppression scale negatively correlates with both PTSD and OCD symptoms, as well as with the intrusion scale (van Schie et al., 2016). Similarly, the Thought Control Ability Questionnaire (Luciano et al., 2005), which assesses perceived capacity to control thoughts, is negatively associated with psychological problems (for review, see Feliu-Soler et al., 2019). These findings suggest that associations between WBSI scores and poorer psychological functioning may reflect not the inherent harmfulness of thought suppression, but rather the difficulty of eliminating intrusions among individuals with these conditions. This conclusion is further supported by a meta-analysis by Stramaccia and colleagues (2021), which linked ineffective suppression in the TNT task to the presence of psychopathology.

A key strength of the TSI-R is that it enables examination of the correlates of thought suppression in relation to its effectiveness. However, the measure is not without flaws: the suppression attempts scale has been criticized for its lower psychometric properties (van Schie et al., 2016). Unfortunately, satisfactorily isolating this scale may be problematic. Suppression attempts are likely to depend both on the demand for suppression—driven by the frequency of intrusive thoughts—and on suppression efficacy. Greater efficacy may increase use because of perceived utility, but at the same time reduce the overall need for suppression as intrusions become less frequent. Analyzing the combined profile of the three scales might therefore yield more informative results.

Despite its limitations, the TSI-R offers a more comprehensive assessment of thought suppression, without a priori assumptions about its effectiveness or adaptiveness. Unfortunately, despite its advantages, the continued use of the WBSI remains standard practice. According to Google Scholar statistics since the publication of the TSI-R (van Schie et al., 2016) to mid-2025, it has been cited only 21 times, whereas the original article introducing the WBSI by Wegner and Zanakos (1994) has been cited about 900 times in the same period. Although some of the citations of the latter stem from critical discussions, the number of studies employing it still exceeds those using the TSI-R (as illustrated above). This disparity may contribute to perpetuating a misconception regarding the harmfulness of thought suppression.

### Treatment approaches for intrusive thoughts

Thought suppression is especially relevant in the clinical context, as recurrent, distressing thoughts are a feature of many forms of psychopathology (D. A. Clark, 2005). Intrusive memories in PTSD, obsessions in OCD, rumination in depression, and worry in anxiety disorders illustrate how such cognitions can cause significant patient suffering. To provide the best possible support for these individuals, it is essential to determine when thought suppression is likely to be adaptive and when it may instead have harmful effects.

Common interventions in treating disorders characterized by intrusive thoughts include confrontation-based approaches involving thoughts and memories that cause patients distress and discomfort. This approach was pioneered, among others, by Freud, who encouraged patients to share traumatic memories (Rubenstein et al., 2024). Contemporary psychodynamic therapy, derived from Freud's work, appears effective (Shedler, 2010), although its efficacy may not always align with rigorous scientific verification. Conversely, cognitive–behavioral therapy, tailored more closely to such scrutiny, has repeatedly demonstrated effectiveness (Kaczkurkin & Foa, 2015; Querstret & Cropley, 2013; Wenzel, 2021). In cognitive–behavioral therapy, patients also confront unwanted and painful thoughts, evaluating their rationality, striving to accept their presence (Dozois & Beck, 2011), or restructuring their associations (e.g., Brewin, 2001).

A number of psychological interventions involve direct contact with painful thoughts, including imagery rescripting, mindfulness meditation, eye movement desensitization and reprocessing, and cognitive processing therapy (Rubenstein et al., 2024; Todd & Branch, 2022), with exposure therapy being the most prominent example. Exposure therapy, a primary form of cognitive–behavioral therapy, systematically elicits unwanted content (Garner et al., 2021). It has been shown to be effective in treating disorders such as generalized anxiety disorder, depression, OCD, and PTSD (Garner et al., 2021; McLean et al., 2022; Taylor et al., 2003), and is recommended by clinical guidelines as a first-line treatment for the latter two (American Psychological Association, 2025; National Institute for Health and Care Excellence, 2005, 2018). The rationale behind exposure therapy is that avoiding unwanted content prevents individuals from assessing its accuracy (i.e., its correspondence with reality) and from achieving habituation, which reduces intense emotional reactions (Dozois & Beck, 2011). Furthermore, avoiding painful memories can hinder their integration, proper contextual and temporal placement, and assimilation with new information (e.g., Brewin, 2001). As long as certain mental content remains associated with a current, perceived threat, it may resurface as a signal of imminent danger. Once this association is weakened or replaced, the content should be muted.

Against this backdrop, thought suppression appears potentially harmful, as it could impede the extinction of disproportionate emotional reactions that underlie personal suffering. Considering that interventions involving deliberate engagement with painful content often reduce its frequency, it seems reasonable that persistent, life-disrupting thoughts should be addressed under the guidance of a clinician trained in such techniques.

Nevertheless, it is premature to deem thought suppression universally harmful. Indeed, In PTSD and OCD, suppression appears ineffective: patients continue to experience intrusions despite attempts to avoid or suppress them—attempts that are themselves part of the diagnostic criteria for these disorders (American Psychiatric Association, 2013). In depression and anxiety disorders, however, the picture is less clear. On the one hand, research indicates impaired suppression performance in the TNT task among individuals with depression and anxiety (Stramaccia et al., 2021), and clinical observations suggest that such patients often attempt to escape unwanted thoughts with limited success (Dunkley & Robichaud, 2022). On the other hand, they may also hold maladaptive metacognitive beliefs that rumination and worry are beneficial (Dunkley & Robichaud, 2022; Watkins, 2022), which could undermine suppression efforts (Fawcett et al., 2015; Hertel et al., 2018).

Moreover, the treatment of rumination and worry can involve strategies that, at least in theory, incorporate elements of thought suppression. For example, worry habits can be addressed by postponing worry to a designated time and place, thereby controlling the cues that trigger it (Dippel et al., 2024). While this approach does not explicitly frame the process as thought suppression, it requires individuals to interrupt worrisome thoughts in the moment and delay them. Similarly, in Rumination–Focused Cognitive Behavioral Therapy, the use of if–then plans establishes new responses to warning signs of rumination (Watkins, 2022). Such plans can involve eliciting alternative thoughts in response to rumination cues, which resembles the thought substitution strategy used in the TNT task.

Processes related to thought suppression may also be embedded in other psychological interventions. It has been suggested that during cognitive reappraisal, used in cognitive–behavioral therapy, patients must inhibit maladaptive interpretations of an event (direct suppression) and replace them with more adaptive ones (thought substitution; Engen & Anderson, 2018). Similarly, Anderson (Anderson et al., 2025) has speculated that fear extinction may partly recruit the same neural mechanisms engaged in direct suppression during the TNT task.

Interestingly, there is an area of psychological practice that suggests it may sometimes be harmful to confront painful content and, conversely, beneficial to disengage from it. In the 1980 s, Critical Incident Stress Debriefing emerged as an intervention for individuals immediately following traumatic events (Mitchell, 1983, as cited in Stileman & Jones, 2023). This intervention aimed to prevent the development of PTSD by discussing the traumatic experience and the reactions to it (Stileman & Jones, 2023). Although the format of debriefing resembles effective interventions used in PTSD therapy, debriefing itself is not recommended by the World Health Organization (2012) or the National Institute of Health and Care Excellence (2018). This stance is based on studies indicating that debriefing was ineffective or even harmful (e.g., Raphael & Meldrum, 1995; Rose et al., 2002). The latest meta-analysis of debriefing studies also failed to confirm its effectiveness (Stileman & Jones, 2023).

There arose a need to develop new interventions for use immediately after a traumatic event to prevent PTSD. Promising techniques, sometimes referred to as cognitive vaccines against traumatic flashbacks, emerged (Holmes et al., 2009, 2010). These interventions are designed to prevent the full consolidation of sensory memory traces of the trauma (Holmes et al., 2009). The first hours after the event appear crucial for memory consolidation (Shadmehr & Holcomb, 1997; Walker et al., 2003). During this time window, engaging in a visuospatial task (such as playing Tetris; Agren et al., 2023; Holmes et al., 2009) can interfere with the consolidation process, as it utilizes cognitive resources also involved in the task. The resulting effect is opposite to debriefing, where consolidation is reinforced. Through such interference, the sensory aspects of the event, which are known to underlie flashbacks (Brewin, 2014), are not fully encoded. At the same time, memory of the factual details of the event and the ability to voluntarily recall these details should be preserved. This allows individuals to still have the ability to describe the event or provide testimony if needed (e.g., Lau-Zhu et al., 2019).

The effectiveness of cognitive vaccines has been confirmed in studies using drastic video excerpts functioning as an experimental analogue of trauma exposure (for meta-analysis see, Asselbergs et al., 2023). In recent years, an increasing number of studies have demonstrated their efficacy in clinical settings among individuals who have actually experienced trauma (e.g., Horsch et al., 2017; Iyadurai et al., 2018, 2020, 2023; Kanstrup et al., 2021; Kessler et al., 2018; Ramineni et al., 2023). As a result of these interventions, a substantial reduction in the frequency of intrusions has been observed, reaching over 60% (Deforges et al., 2022; Kessler et al., 2018). Nevertheless, further research is needed, since both the theoretical assumptions and the long-term effectiveness of this intervention are not always supported, as shown in a recent multilab replication study (Wessel, Krans, et al., 2025).

The encouraging data on cognitive vaccines and the ineffectiveness or even harmfulness of debriefing suggest that after a traumatic event, better outcomes are achieved by not thinking about the experience and redirecting attention to other content. Psychological practice, therefore, indicates that the adaptiveness of thought suppression may depend on the context: whether the suppression involves highly intrusive and frequent thoughts typical of PTSD and OCD, or thoughts about a recently experienced event whose memory trace has not yet consolidated. Against this background, general recommendations not to suppress thoughts should be nuanced with references to the specific context of the suppression and likely other factors as well.

Relativizing the adaptiveness of thought suppression in the clinical context also opens the way for research into its deliberate use and enhancement as a means of better managing intrusive thoughts. Not all patients benefit from available therapeutic methods, and a considerable proportion discontinue treatment—particularly exposure therapy, which is highly distressing (Rubenstein et al., 2024). Even where treatment is effective, it often does not lead to a complete elimination of symptoms (e.g., Rubenstein et al., 2024; Sündermann & Veale, 2022; Y. Wang et al., 2024). Unwanted thoughts may still recur, despite habituation and contextual placement, causing distress. Likewise, individuals without psychiatric disorders may also experience intrusive thoughts. Theoretically, in such cases, the ability to suppress thoughts could be not only harmless but even beneficial.

Therefore, alongside established methods such as cognitive–behavioral therapy, it is worth developing new techniques that deliberately engage thought suppression. Current research is exploring methods to support suppression (Chen & Gao, 2023; Hertel & Calcaterra, 2005; Hertel & McDaniel, 2010; Joormann et al., 2009) or to facilitate the forgetting of subliminally presented unwanted material through the amnesic shadow mechanism (Zhu et al., 2022). Furthermore, assuming that thought suppression is, to some extent, already embedded within existing psychological interventions, their efficacy might be improved by strengthening this suppression component. The development of such methods will depend on further studies that specify the conditions under which thought suppression is adaptive.

### Experimental and quasi-experimental investigations into the adaptiveness of thought suppression

To investigate the factors that condition the adaptiveness of thought suppression, further experimental studies are necessary. The adaptiveness of suppression can be examined by ensuring the ecological validity of the stimuli used. Experiments have emerged where participants were required to suppress thoughts about aversive images (the TNT paradigm; e.g., Depue et al., 2007; Gagnepain et al., 2017; Harrington et al., 2021; Nishiyama & Saito, 2022), such as drastic film excerpts (a combination of the trauma film and the WB paradigms; Davies & Clark, 1998; Nixon et al., 2009; Oulton et al., 2016), pain stimuli (the WB paradigm; Cioffi & Holloway, 1993; Kreddig et al., 2022), personal memories (the TNT paradigm; e.g., Noreen & Macleod, 2013; Satish et al., 2022, 2024), or content resembling intrusive thoughts (the TNT paradigm; Benoit et al., 2016; the WB paradigm; Corcoran & Woody, 2009; Malinowski et al., 2019). For instance, Benoit et al. (2016), using the TNT procedure, examined the consequences of suppressing imaginings about feared events. The suppressed events were not only remembered worse but also elicited less anxiety than baseline events. Similarly, Satish et al. (2022, 2024) demonstrated that suppressing memories of one’s immoral behaviors led to a reduction in intrusion frequency and weakened negative emotions associated with the memory. Generally, the capacity to suppress emotionally laden thoughts in the TNT task and consequently alleviate negative emotions has been demonstrated quite consistently, using behavioral (e.g., Satish et al., 2024), physiological (e.g., Harrington et al., 2021), and neuroimaging measures (e.g., Gagnepain et al., 2017).^2^

The effectiveness of thought suppression during laboratory experiments, while suggestive, cannot conclusively determine the adaptiveness of this strategy in everyday life. To investigate this, it is necessary to examine the impact of thought suppression on an individual's mental health. This issue is usually addressed in correlational studies, which, by their nature, cannot determine causality and often have notable methodological flaws (as discussed earlier). To the best of my knowledge, only one experimental study has explored the impact of suppressing personally significant thoughts on the psychological functioning of participants.

In this study, Mamat and Anderson (2023) asked each participant to create a list of events that could potentially occur in the future. Using an adapted TNT procedure, participants practiced suppressing imaginings of selected negative or neutral events over 3 days. Consistent with previous research, immediately after the training, the memory for suppressed events was poorer, and the emotional response weaker compared to the memory and emotions associated with baseline events. Participants also showed overall improvement in mental health compared to their pretraining state, with greater improvement observed in those who suppressed fearful events rather than neutral ones. For example, suppressing fearful episodes reduced the risk of decreased well-being and worsening depression symptoms by approximately 50% compared to the group suppressing neutral episodes.

Three months later, individuals who trained to suppress fearful episodes showed further improvement in depressive symptoms, while those in the group suppressing neutral episodes experienced improvement in worrying scores. Examining mental health indicators both immediately after training and three months later, anxious individuals and those with higher scores on the PTSD scale benefited the most from learning thought suppression, but only in the group that suppressed fearful events. The predictors of improved mental health were the extent to which participants were able to weaken negative emotions associated with the suppressed fearful events and the frequency of thought suppression during the three months following the training.

The results of this study are encouraging and point towards the potential adaptiveness of thought suppression. In a series of analyses conducted by the study authors, no traces of paradoxical effects were found. There is a need for further experimental research examining the impact of thought suppression on individuals' psychological functioning. However, as highlighted by Mamat and Anderson (2023), obtaining ethics approval for such studies is challenging. If thought suppression is a priori considered maladaptive, asking participants to utilize this strategy in natural settings becomes ethically questionable. Meanwhile, as I have attempted to demonstrate in the article, negative beliefs about the effectiveness of thought suppression have uncertain empirical support.

Of course, drawing strong conclusions about the adaptiveness of thought suppression based on one study would be unreasonable. However, the issue can be approached from yet another perspective by examining the consequences of thought suppression inability. Individuals vary in their ability to suppress thoughts (Levy & Anderson, 2008). Particularly, individuals suffering from psychiatric disorders such as generalized anxiety disorder, depression, or PTSD face difficulties in thought suppression (e.g., Catarino et al., 2015). For instance, meta-analysis of studies using the TNT procedure demonstrated compromised ability to effectively forget suppressed thoughts among clinical and subclinical samples (Stramaccia et al., 2021). These findings align with a broader body of research on impaired cognitive control in mental disorders, manifested, for example, in a reduced ability to remove no longer relevant material from working memory (for meta-analysis, see Zetsche et al., 2018) and in poorer memory control in the directed forgetting task (for meta-analysis, see Pevie et al., 2023).

It is plausible that difficulty in thought suppression contributes to the development of certain disorders, such as PTSD. For instance, Streb and colleagues (2016) demonstrated that the magnitude of suppression-induced forgetting predicted discomfort caused by intrusions following exposure to drastic film excerpts. Moreover, Mary and colleagues (2020) showed that during thought suppression, the brains of individuals with PTSD behave differently than the brains of participants who were also exposed to trauma but did not exhibit PTSD symptoms. In the latter group, thought suppression in the TNT task was effective and accompanied by coupling between prefrontal regions involved in cognitive control and memory regions associated with recalling mental content. In contrast, in the PTSD group there was no such coupling observed and—what apparently follows—thought suppression failed.

In summary, existing studies suggest that thought suppression may be adaptive in non-clinical populations, whereas clinical populations appear to have a more limited capacity to use this strategy effectively. Nevertheless, the research paradigms employed so far have notable limitations. While some experiments have examined more ecologically valid contents, such as autobiographical memories (e.g., Noreen & Macleod, 2013) or worries (e.g., Benoit et al., 2016), the majority still rely on simple stimuli and highly controlled laboratory conditions. Studies investigating the suppression of complex, multisensory material in more naturalistic contexts are lacking (Fawcett et al., 2024). Furthermore, current paradigms make it difficult to assess the outcomes of the long-term, habitual, and unconscious blocking of unwanted thoughts. For example, the TNT paradigm—originally designed as a tool for the empirical study of Freudian repression (Anderson & Green, 2001; Erdelyi, 2006; cf. Boag, 2017)—only allows a limited examination of blocking access to autobiographical memories (Wessel, 2024).

Continued development of research on thought suppression would be highly beneficial. Nevertheless, available data already provide grounds for optimism regarding the adaptive use of thought suppression. Thus, beyond enhancing measures of thought suppression, attention should be directed toward identifying the contexts in which it functions adaptively and those in which it may have detrimental consequences.

### Desirable and undesirable outcomes of thought suppression

Fortunately, in searching for the conditions under which thought suppression is adaptive, we do not have to start from scratch. Existing findings on the adaptiveness of forgetting provide valuable guidance. Thought suppression and forgetting can be seen as related processes, particularly when considering suppression and intentional forgetting in working memory. Moreover, experiments using the TNT paradigm suggest that suppression can also lead to forgetting over longer time spans. Consequently, at least some situations in which forgetting is adaptive should overlap with those in which suppression is beneficial.

Among publications highlighting the functions of forgetting (e.g., Bjork, 1989; Nørby, 2015, 2018; Schacter et al., 2011), the comprehensive paper by Fawcett and Hulbert (2020) is particularly relevant to thought suppression. The authors devote considerable attention to retrieval suppression—preventing unwanted thoughts from entering consciousness. Several of the functions of forgetting they describe align with potential functions of suppression. For example, mood and motivation regulation can be achieved either through the lasting weakening of memories of negative events or through the real-time suppression of such thoughts. Likewise, maintaining interpersonal relationships may be supported by the long-term forgetting of grievances or awkward incidents, as well as by suppressing thoughts about—perhaps mistakenly assumed—ill intentions of others. Organizing information by forgetting redundant details and outdated or currently inappropriate data could also be achieved by suppressing thoughts about information judged irrelevant or false. Other parallels include maintaining psychological stability by forgetting information inconsistent with one’s self- and world-views, facilitating change by forgetting previous ways of acting, and supporting creativity by forgetting conventional solutions.

Whether these functions are fulfilled optimally or in a distorted way depends on context. For instance, a complete absence of thoughts about distressing events may improve mood, but at the cost of losing contact with reality. Similarly, one should not always assume good intentions or block out information that conflicts with existing beliefs. Such complete suppression could leave a person unprepared for real threats or prevent them from learning from mistakes. The same caution applies to information organization or creative problem-solving, where it is rarely possible to know in advance which content will be useful. Thus, deciding what is worth suppressing or forgetting inevitably involves uncertainty.

A possible wrong decision about which contents to suppress should have milder repercussions when suppression is applied temporarily, for a short period, or in a specific situation. For example, if faced with accusations contrary to our self-knowledge, we might temporarily suppress these thoughts to focus on defending our reputation. This could be an effective strategy when the accusations are false and meant to destabilize us. However, if it turned out that we had done something wrong, it would be worth reflecting on it—even if only after the incident. This could foster personal growth and facilitate the repair of relationships. Conversely, deciding never to think about such accusations would remove that opportunity.

There are also cases where long-term suppression, leading to poorer memory of certain content, may be adaptive—such as suppressing thoughts about a social blunder or a mistake that has already been understood and corrected. Similarly, overly self-critical thoughts or unrealistic worries typically do not serve adaptive purposes and instead impair functioning. A safeguard against the potential downsides of long-term suppression could be to reflect on them and make a deliberate decision as to which is likely to be more beneficial: suppressing or thinking. General guidelines indicating when suppression is typically helpful and when it is likely to be detrimental would be invaluable.

The adaptiveness of thought suppression may depend not only on the content being suppressed, but also on who is doing the suppressing. As noted, individuals differ considerably in their ability to suppress thoughts (Levy & Anderson, 2008). Those who find suppression easy may benefit from it, but risk overusing it to avoid inconvenient truths (e.g., Hayes et al., 1996; Myers, 2010). On the other hand, when suppression fails, it can lead to frustration and negative emotions (e.g., Kelly & Kahn, 1994; Purdon et al., 2005, 2011), particularly in people with maladaptive metacognitive beliefs such as the importance of control thoughts or thought–action fusion (Sandstrom et al., 2024; Shemrani et al., 2025). Individuals who continue to experience intrusive thoughts despite suppression—such as patients with PTSD or OCD—may be better off avoiding this self-regulation strategy altogether.

Research is underway on how to facilitate suppression when it does not succeed spontaneously. Some studies suggest that suppression via thought substitution can help those unable to eliminate unwanted thoughts in other ways (e.g., Joormann et al., 2009). Such techniques could also assist individuals who normally suppress effectively but are hindered by temporarily reduced cognitive resources. WB studies show that cognitive load impairs suppression and may trigger the immediate enhancement effect (for meta-analysis, see D. A. Wang et al., 2020). TNT studies similarly indicate that successful suppression requires specific cognitive resources, as evidenced, for example, by worsened suppression in participants under cognitive load (Noreen & De Fockert, 2017), fatigued from a prolonged TNT phase (van Schie & Anderson, 2017), or experiencing sleep deficits (Harrington et al., 2021).

If suppression draws on cognitive resources, it raises the question of the costs of long-term use. Poorer suppression in people with mental disorders may partly reflect the fatigue of constant psychological struggle (Brewin, 2001; van Schie & Anderson, 2017). A hypothesis worth testing is whether prolonged thought suppression may lead to a further reduction in cognitive control capacity. This would imply the adaptiveness of suppression may depend on managing cognitive resources in a way that prevents overtaxing them. In such cases, short-term thought suppression would likely be safer than prolonged suppression.

Thus, thought suppression may have side effects of taxing cognitive capacity. Unfortunately, other undesirable consequences are also possible. TNT research shows the amnesic shadow effect—poorer memory for material presented near the time of suppression (Hulbert et al., 2017). Moreover, while participants often recall think items better than baseline items after the TNT phase (Anderson & Huddleston, 2012), this is not always the case (for review, see Nardo & Anderson, 2024). Dalgleish and colleagues (2008) also suggest that suppression of memories can reduce autobiographical memory specificity overall. Thus, attempting to suppress one thought may come at the cost of forgetting content one would prefer to retain. On the other hand, suppression may also contribute to the recall of other unwanted thoughts, as when, in a low mood, avoiding one thought leads to thinking about other mood-congruent—and therefore more accessible—contents (Dalgleish et al., 2008).

Hypothetically, thought suppression may also confer unintended benefits. Hulbert and Anderson (2018) examined TNT performance in healthy participants differing in the extent of prior aversive experiences. Individuals with more traumatic history exhibited greater suppression-induced forgetting. The authors interpreted this finding as reflecting repeated engagement in suppression across the lifespan, effectively serving as incidental training. Consequently, these individuals appeared to develop an enhanced capacity to block intrusions, thereby fostering resilience.

Numerous factors may influence whether thought suppression yields desirable or undesirable outcomes, and many questions remain unresolved. Much of the discussion above is necessarily speculative, and definitive conclusions will require focused empirical investigation. Importantly, such research can only move forward if we first recognize that thought suppression may, under certain conditions, be adaptive.

## Conclusions

The aim of this paper was to address the question of the effectiveness and adaptiveness of thought suppression. Conclusions regarding effectiveness were drawn from experimental evidence, while those concerning adaptiveness were based on questionnaire, experimental, and quasi-experimental studies, as well as clinical practice.

With respect to suppression effectiveness, research using two thought suppression paradigms indicates that healthy individuals are capable of successfully suppressing thoughts in the short term—that is, during active suppression efforts. Findings regarding post-suppression effects, however, are more divergent: while research in the TNT paradigm supports suppression effectiveness over longer time frames, studies using the WB paradigm are known for the paradoxical rebound of the suppressed thought. This apparent contradiction can be resolved by considering the replicability of each paradigm’s effects. The suppression-induced forgetting effect, characteristic of TNT research, replicates reliably, whereas the WB task’s rebound effect is often absent and likely influenced by publication bias. Therefore, paradoxical effects of thought suppression are not convincingly demonstrated. Overall, available evidence suggests that thought suppression can be effective in healthy individuals—especially in the short term, but seemingly also in the long run.

Answering the question of adaptiveness is more challenging. Questionnaire-based studies exploring links between the tendency to suppress and the presence of mental health problems have, for the most part, failed to distinguish between the frequency of suppression attempts and the frequency of intrusions. As a result, correlations found in these studies are best interpreted as evidence that people with mental disorders experience more frequent intrusions. When intrusion frequency is separated from suppression frequency, associations between psychopathology and suppression are usually non-significant. Moreover, some studies have found that the ability to suppress effectively is negatively related to symptoms of disorders such as PTSD and OCD (e.g., van Schie et al., 2016).

At the same time, individuals with these disorders often attempt to suppress their intrusive thoughts—typically without success. Unsurprisingly, treatment for such problems focuses on confronting unwanted thoughts in order to address their root causes, which may include maladaptive beliefs or the blocking of habituation and fear extinction. Cognitive–behavioral therapy, especially with exposure techniques, is based on these principles, has established effectiveness, and should remain a standard treatment for disorders involving intrusive thoughts.

However, this does not mean that engaging with unwanted thoughts is the only viable coping strategy. For example, immediately after a traumatic event, it may be preferable to occupy cognitive resources with a task that blocks trauma-related thoughts and hinders full consolidation of traumatic memories (e.g., Holmes et al., 2009). Such an approach can be more effective at preventing PTSD than debriefing, which involves narrating the recently experienced trauma. Thus, the most adaptive approach may depend on whether trauma-related thoughts appear immediately after the event or, as in PTSD, persist over a long period.

The adaptiveness of thought suppression may depend not only on the timing of unwanted thoughts but also on who experiences them. People differ in their suppression abilities (Levy & Anderson, 2008), with clinical populations often showing particular difficulty (Stramaccia et al., 2021). If patients with certain disorders cannot suppress thoughts effectively, it may seem that suppression is unhelpful or even harmful. However, such a conclusion is too far-reaching—clinical observations cannot determine the adaptiveness of suppression across the general population (see also Fawcett & Hulbert, 2020). In fact, initial studies on suppression training in healthy participants suggest beneficial outcomes (Mamat & Anderson, 2023).

Reports of ironic effects of thought suppression emerged early in the study of mental control, and these align with a presumed cognitive bias toward noticing and remembering failures of self-control more readily than successes. By its nature, successful thought suppression is forgotten—its success lies in forgetting the suppressed content. Such factors have likely reinforced the widespread belief in the inefficacy and maladaptiveness of suppression. Nevertheless, as I have sought to demonstrate, thought suppression can be both effective and adaptive.

Future research should focus on identifying the precise conditions of the effectiveness and adaptiveness of suppression. Open questions include: the distribution of suppression abilities across the population; ways to support those who struggle with suppression; the long-term effects of suppression; and the outcomes of suppression training. At the same time, future studies should avoid the methodological pitfalls of earlier work. Addressing these questions could provide the basis for developing effective strategies to help people manage the common problem of intrusive thoughts.

### Author Note

This work was supported by the National Science Centre (Poland) under Grant 2019/33/N/HS6/02568.

## Data Availability

Not applicable.

## Footnotes

1 In the meta-analysis by Magee and colleagues (2012), data on effect sizes in individual studies were not included, therefore, I do not provide the proportion of rebound effect occurrence in the studies analyzed by the authors.

2 Studies in the WB paradigm yield inconsistent results, sometimes indicating emotional benefits from thought suppression (Kreddig et al., 2022; Malinowski et al., 2019; Oulton et al., 2016), and other times pointing to its negative effects (Cioffi & Holloway, 1993; Corcoran & Woody, 2009; Davies & Clark, 1998). Due to the poor replicability of this paradigm, I do not discuss these studies in detail.
